# XMU-MP-1, the Hippo Signaling Pathway MST-1 Kinase Inhibitor, Prevents the Development of Drug Resistance to Doxorubicin in Hematological Tumor Cells

**DOI:** 10.3390/ph19071075

**Published:** 2026-07-12

**Authors:** Alexander G. Stepchenko, Elizaveta V. Pankratova

**Affiliations:** Engelhardt Institute of Molecular Biology, Russian Academy of Science, Moscow 119334, Russia

**Keywords:** drug resistance, Hippo pathway, XMU-MP-1, cell cycle, anthracycline

## Abstract

**Background/Objectives**: The search for new drugs which could suppress drug resistance development in tumor cells is extremely important for clinical practice. Inhibitors of cell signaling pathways that control cell proliferation and death can be used in complex therapy of malignant tumors. **Methods**: Cell cycle assay using flow cytometry, In Vitro Cell Viability Assay Cell chemosensitivity was analyzed by direct cell counting after trypan blue staining using a microscope. **Results**: In the present work, we have shown that the combined action of doxorubicin and XMU-MP-1, the MST1/2 kinase inhibitor in the Hippo signaling pathway, prevents drug resistance development in Namalwa cells, significantly slows drug resistance development in K562 cells, and restores the sensitivity of resistant K562 cells to doxorubicin. We have shown that compared to monotherapy, the combination of doxorubicin and XMU-MP-1 causes a significant decrease in cell division rate and cell death in hematological tumor cells such as Burkitt’s lymphoma Namalwa and chronic myeloid leukemia (CML) K562 cells. Cell cycle analysis revealed that the combined action of XMU-MP-1 and doxorubicin caused a catastrophic cell cycle disruption and a significant increase in the number of cells undergoing apoptosis containing fragmented DNA. **Conclusions**: XMU-MP-1 can be potentially used in combination with anthracyclines for the treatment of hematological malignancies and, in particular, drug-resistant cancer types.

## 1. Introduction

Malignant tumors of hematopoietic and lymphoid tissue can be divided into two large groups—lymphomas and leukemias. Many of them are characterized by unfavorable prognosis and low survival rate.

Doxorubicin is used to treat many different malignancies including lymphoblastic leukemias and non-Hodgkin’s lymphomas and is often considered as the first-line drug. Its activities include DNA intercalation, topoisomerase II inhibition, and reactive oxygen species generation, all of them ultimately leading to DNA damage, cell cycle disturbance, apoptosis, and cell death. Although this drug is highly efficient, its use is often associated with significant side effects, such as high cardiotoxicity, necrotizing colitis, and reduced blood cell count, as well as acquired drug resistance. Hence, it is clinically important to find ways to reduce the effective therapeutic dose of doxorubicin and prevent drug resistance development.

Higher efficiency and selectivity of chemotherapy drugs can be achieved by combining them with compounds that modulate the functioning of cell signaling pathways. Drugs that modulate the Hippo signaling pathway are considered to have high potential for the combination therapy of cancer [1].

The Hippo signaling pathway is an evolutionary conserved signaling cascade that regulates many biological processes. The Hippo signaling pathway core in mammals consists of the kinase cascade including the MST1 (serine-threonine kinase 4 (STK4)) and MST2 (serine-threonine kinase 3 (STK3)) kinases and the LATS1 and LATS2 kinases, as well as the downstream effectors—the YAP and TAZ transcription coactivators. These key components of the Hippo pathway control transcriptional programs involved in cell proliferation, survival, and motility, stem cell maintenance and cell differentiation along with tissue regeneration and organ growth, performing complex regulation through the formation of biomolecular condensates [2].

Tumor cells with impaired Hippo signaling regulation not only overcome the internal cell death mechanisms, but also show resistance to chemotherapeutic drugs or targeted molecular therapy, the latter being another factor contributing to cancer recurrence [3]. Unlike epithelial tumors, genetic inactivation of MST1 or high YAP expression leads to the inhibition of tumor growth and activation of apoptosis in hematological tumors [4].

XMU-MP-1 (4-((5,10-dimethyl-6-oxo-6,10-dihydro-5H-pyrimido [5,4-b]thieno [3,2-e][1,4]diazepin-2-yl)amino)benzenesulfonamide), the potent reversible selective inhibitor of the mammalian sterile 20-like kinases 1 and 2 (MST1/2), the key molecules in the Hippo signaling pathway, has been described. XMU-MP-1 activates the downstream effector, the Yes-associated protein (YAP), by blocking the activity of the MST1 and MST2 kinases [5,6].

With the half-life period of 1.2 h and bioavailability of 39.5%, XMU-MP-1 has demonstrated outstanding pharmacokinetics in rats. Maximum YAP inhibition is achieved between 1.5 and 6 h after intraperitoneal administration of the drug (1–3 mg/kg) [6]. Preclinical studies of XMU-MP-1 have shown the absence of toxic effects when administered in the suggested therapeutic dose and in a four-times higher dose [6,7,8,9].

Therapeutic effects of MST1 kinase activity suppression have been described. The role of XMU-MP-1 in the treatment of diabetes, osteoarthritis, obesity, liver, and intestine pathologies has been demonstrated. It has also been shown to improve the survival of cardiomyocytes and stimulate platelet recovery after immune thrombocytopenia [7,8,9,10,11,12,13]. Thus, XMU-MP-1 can be used in clinical practice if a justified method of application could be suggested for it.

We have previously demonstrated that XMU-MP-1 inhibits the growth of human hematological tumor cells by blocking the cell cycle and inducing apoptosis [14].

In the present study, we used two human tumor cell lines including the doxorubicin-sensitive/resistant K562 chronic myeloid leukemia cell line and the Burkitt’s B-cell lymphoma cell line Namalwa to investigate whether XMU-MP-1 can inhibit drug resistance development in tumor cells and reverse acquired drug resistance. These cancer cell lines were used because chronic myeloid leukemia (CML) is one of the most common malignant hematological neoplasms, and Burkitt’s lymphoma is one of the most aggressive tumors. Both of them are difficult to treat using chemotherapy and are known to develop drug resistance quite rapidly. In the present work, we have found that XMU-MP-1 is able to inhibit the development of doxorubicin drug resistance in Namalwa cells, prevent the induction of doxorubicin resistance in K562 line, and increase the sensitivity of cancer cells, in particular, resistant cancer cells to doxorubicin in vitro.

## 2. Results

### 2.1. XMU-MP-1 and Doxorubicin Arrested Cell Cycle and Induced Apoptosis in Hematological Tumor Cells

It is well-known that treatment with low doses of doxorubicin causes mitotic catastrophe in the cells [10,11]. We found that XMU-MP-1 also causes mitotic catastrophe, which is characterized by cell cycle arrest and reveals unique nuclear morphology featuring micro- and/or multi-nuclei. Microscopic examination also revealed many apoptotic changes, including nuclei fragmentation, cell swelling, and noticeable changes in the cell size—from cell fragments to large swollen cells. Co-treatment of cells with the MST1 kinase inhibitor XMU-MP-1 and doxorubicin further increased the number of cells with mitotic catastrophe morphology demonstrating the above-mentioned cell morphology abnormalities (Supplementary Materials Figure S1). The normal morphology of these cells could not be restored by transferring them into the fresh medium, and they continued to die. This motivated us to investigate how these two drugs affect the cell cycle separately and in combination. We have previously shown that XMU-MP-1 induces cell cycle arrest at the G2/M phase and activates genes that regulate the programmed cell death in the Namalwa cells [14].

**Figure 1:**
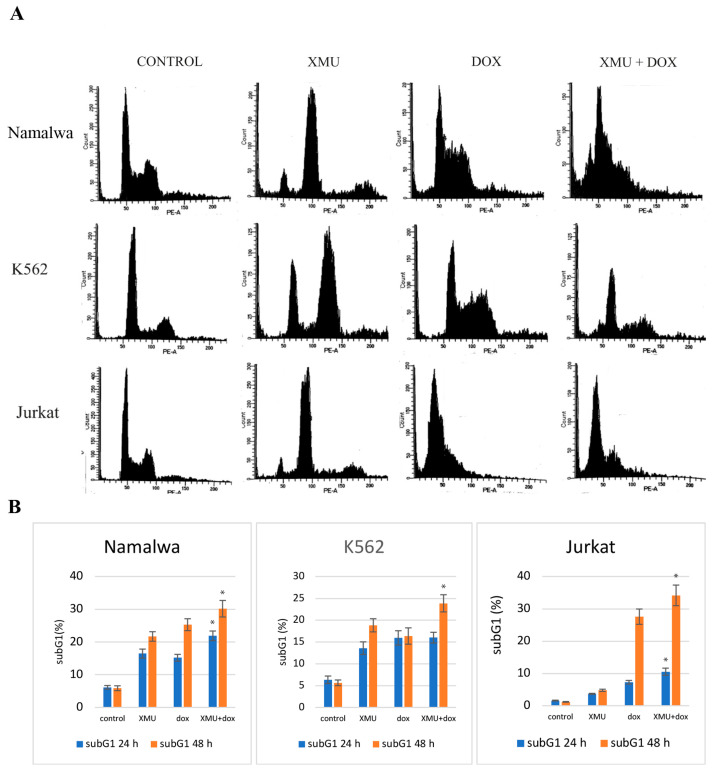
Effects of XMU-MP-1 and doxorubicin on cell cycle and apoptosis. (**A**) Flow cytometry analysis in the Namalwa, K562, and Jurkat cells. Cell cycle phase distribution across the cell cycle phases obtained by PI staining of non-treated cells (only DMSO) and cells treated with 1.25 µM XMU-MP-1, or with 0.6 µM doxorubicin, or with 1.25 µM XMU-MP-1 + 0.6 µM doxorubicin for 24 h. (**B**) Sub-G1 quantification. Results of cell cycle analysis in the Namalwa, K562, and Jurkat cells showing mean percentage of cells in the sub-G1 phases, apoptotic cells. Bar height reflects the mean percentage. Plots show the mean ± SEM for three independent biological experiments. *t*-tests were performed and asterisks indicate significant difference compared to monotherapy (* *p* < 0.05).

We assessed the effects of XMU-MP-1 and doxorubicin on cell cycle in the Namalwa, K562, and Jurkat lines using flow cytometry. The results showed that treatment with XMU-MP-1 or doxorubicin individually arrested the cell cycle in these cells at the G2/M and G1/S phases, respectively (Figure 1B). We demonstrated that the combined treatment with XMU-MP-1 and doxorubicin further enhanced the pathological cell cycle changes in all three cell lines. Moreover, the treatment of the K562, Namalwa, and Jurkat cells with XMU-MP-1 in combination with Dox for 24 or 48 h led to an increased subG1 peak, which implies that combined therapy induced stronger apoptosis than monotherapy (Figure 1B).

### 2.2. XMU-MP-1 Prevents Doxorubicin Drug Resistance Development in the Namalwa and K562 Cells

We have previously shown that XMU-MP-1 specifically affects cell death in the hematopoietic tumor cells such as Namalwa, K562, Jurkat, and others [14].

We set out to investigate whether treatment with XMU-MP-1 could prevent the development of doxorubicin resistance. We treated the Namalwa and K562 cells with doxorubicin at the concentrations of 0.0375; 0.075; 0.15; 0.3; 0.6, and 0.9 µM, and with XMU-MP-1 at the concentrations of 0.625; 1.25, and 2.5 µM, and performed long-term observations of the cells exposed to each compound individually and to the combination of doxorubicin and XMU-MP-1.

We found that cell death rate and cell death pattern both strongly depended on doxorubicin and XMU-MP-1 application regimen, as well as on the cell line.

The most pronounced cell population growth suppression effect was achieved in the Namalwa and K562 cells when they were pre-cultivated in the presence of XMU-MP-1 before the addition of doxorubicin.

For the Namalwa cells, the strongest cytotoxic and cytostatic effect was achieved when cells were pre-incubated with XMU-MP-1 for 2 days, with subsequent treatment with doxorubicin after removing XMU-MP-1. In this case, cells died at much higher rate than when they were cultured in the presence of XMU-MP-1 and doxorubicin together.

**Figure 2:**
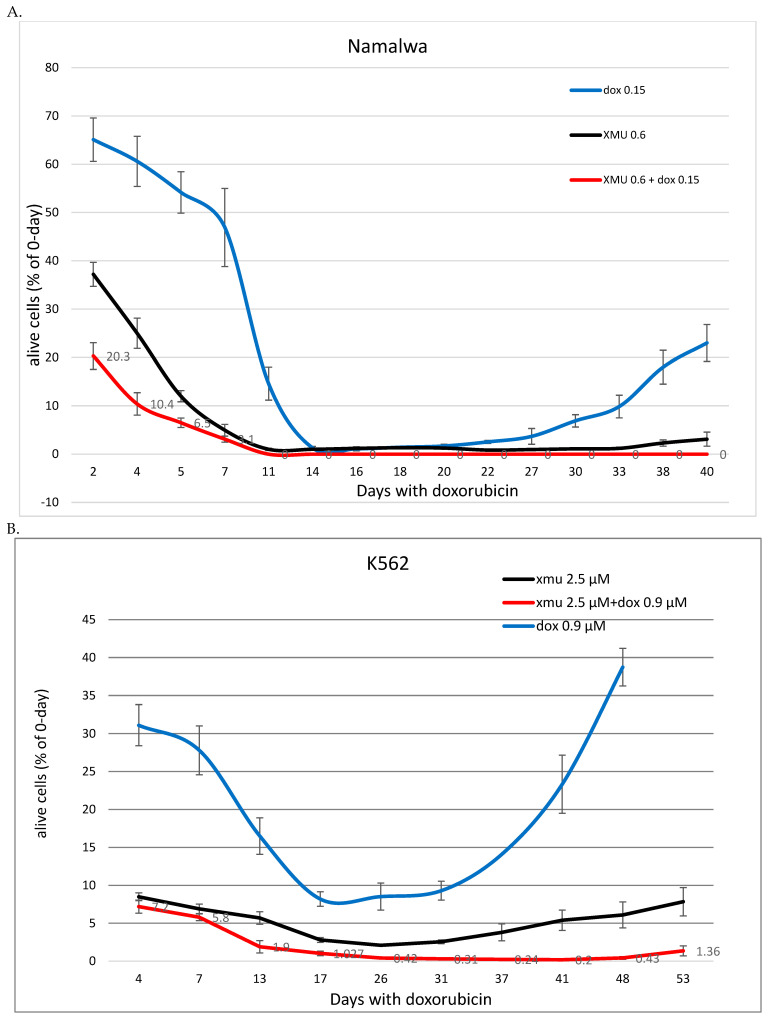
Pretreatment with XMU-MP-1 prevents the development of doxorubicin drug resistance in the Namalwa and K562 cells. (**A**) Namalwa. (XMU 0.6 + dox 0.15)—Namalwa cells were incubated for 2 days in the medium containing 0.6 µM XMU-MP-1, then cells were pelleted and transferred to the medium containing 0.15 µM doxorubicin; (XMU 0.6)—cells were incubated in the medium containing 0.6 µM XMU-MP-1, and (dox 0.15)—cells were incubated in the medium containing 0.15 µM doxorubicin. (**B**) XMU-MP-1 slows the development of drug resistance to doxorubicin in the K562 cells. (XMU 2.5 + dox 0.9)—K562 cells were incubated for 4 days in the medium containing 2.5 µM XMU-MP-1, then cells were incubated in the medium containing 2.5 µM XMU-MP-1 + 0.9 µM doxorubicin; (XMU 2.5)—cells were incubated in the medium containing 2.5 µM XMU-MP-1, and (dox 0.9)—cells were incubated in the medium containing 0.9 µM doxorubicin. The plots show % of live cells relative to the 0-day control. Mean ± SEM for three independent experiments are presented. *p*-values (Supplementary Materials Table S1) were calculated using Welch’s *t*-test for the Namalwa and K562 cell groups treated with (XMU-MP-1 + DOX) or only XMU-MP-1 or DOX.

We have demonstrated that pretreatment of the Namalwa cells with XMU-MP-1 suppressed the development of drug resistance to doxorubicin and led to 100% cell death by around days 11–16 of cultivation (Figure 2A). This effect was the most pronounced at the XMU-MP-1 concentration of 0.6 µM and doxorubicin concentrations of 0.15 and 0.3 µM, that is, at the XMU-MP-1:Doxorubicin ratio of approximately 4:1–2:1.

Monotherapy with doxorubicin at the concentrations of 0.15 and 0.3 µM caused, at first, considerable cell death. However, after about 14–20 days, secondary growth of the cell population began and progressive development of drug resistance could be observed. In the case of the XMU-MP-1 monotherapy, Namalwa cells died at high rate, and by day 11–14, not more than 1% of the original cell population remained (from day 0). Nevertheless, after about 25–30 days, the cell population began to increase in size, which meant that development of drug resistance started. When cells were treated with XMU-MP-1 for only 48 h, washed and further cultured in the fresh medium, then after a short period of cell death, the cell population started to expand and by day 9 reached approximately 45–50% of the initial population at day 0.

The K562 cells die at much higher rate if doxorubicin is added after pre-cultivation in the medium containing XMU-MP-1, and cells are further cultured in the XMU-MP-1 + doxorubicin medium.

Maximum doxorubicin resistance suppression effect was achieved in the K562 cells by pretreating them with XMU-MP-1 for 3–5 days and subsequently culturing them in the XMU-MP-1 + doxorubicin medium. After 26 days, less than 0.8% of the initial cell population remained alive (control—day 0 of the experiment), and by day 54, just few live cells were still present. Cells stopped dividing, but died very slowly. For the K562 cells, doxorubicin concentrations of 0.6 and 0.9 µM (0.348 µg/mL and 0.522 µg/mL, respectively) and XMU-MP-1 concentration of 2.5 µM were the most effective in the combined use, which implies XMU-MP-1:Doxorubicin ratio of approximately 4:1–3:1. In the case of doxorubicin monotherapy, the cells died rapidly at first, but after about 14–20 days, drug resistance to doxorubicin started to develop, and the number of cells began to increase slowly. In the case of XMU-MP-1 monotherapy, cells died rapidly; however, after about 20–25 days, XMU-MP-1-resistant cells started to appear (Figure 2B).

It is well-known that when K562 cells are cultured in the presence of doxorubicin, they develop multi-drug resistance phenotype rather quickly [15]. Our results indicate that XMU-MP-1 significantly delays drug resistance formation in the K562 myeloblastic leukemia line cells, and also prevents the formation of drug resistance in the Burkitt’s B-cell lymphoma line Namalwa.

### 2.3. XMU-MP-1 Restores the Sensitivity of the K562 Cells to Doxorubicin

To test whether XMU-MP-1 could restore the sensitivity of drug-resistant cells to a cytostatic, we induced resistance to doxorubicin in the K562 cell line by growing cells in the presence of incremental concentrations of doxorubicin (37–600 nM), increasing doxorubicin concentration every two weeks during 60 days [15]. Doxorubicin sensitivity of the obtained sublines was measured in the MST assay and by direct counting of live cells under the microscope. Long-term growth in the presence of doxorubicin increased the resistance (IC (50)) of K562 cells in a concentration-dependent way. Two doxorubicin-resistant K562 sublines (DR1 and DR2) (600 nM) and non-resistant original cells (NoDR) were used for further work.

Doxorubicin-resistant K562 cells (DR1 and DR2) were cultured in the medium containing 0.6 µM doxorubicin for 30 days. Cells were then washed to remove the doxorubicin-containing medium, and were inoculated into the medium containing either XMU-MP-1, or 0.9 µM doxorubicin, or XMU-MP-1 + 0.9 µM doxorubicin, or the DMEM medium with only DMSO, cultured for 8 days, and the number of live cells was then counted. The parental cells with no drug resistance (NoDR) were cultured under the same conditions. Upon 8 days of culturing, live cells were counted under the microscope following trypan blue staining. The percentage of live cells in each group was calculated relative to the same cell group cultivated without the addition of XMU-MP-1 or doxorubicin.

The positive effect of XMU-MP-1 on doxorubicin resistance suppression was additionally confirmed by the results of sensitivity tests performed for the original (parental) (No DR) and resistant (DR1 and DR2) cells lines (Table S2). We observed a 13-fold and 16-fold increase in doxorubicin resistance in the DR1 and DR2 lines: IC50 = 0.52 µM in the case of the sensitive K562 cells (No DR), and IC50 = 6.84 µM (DR1) and IC50 = 8.69 µM (DR2). At the same time, co-incubation with 2.5 µM XMU-MP-1 significantly increased cell sensitivity to doxorubicin: IC50 = 0.22 µM (No DR), and IC50 = 0.52 µM (DR1) and IC50 = 0.61 µM (DR2) in the case of the resistant cells.

**Figure 3:**
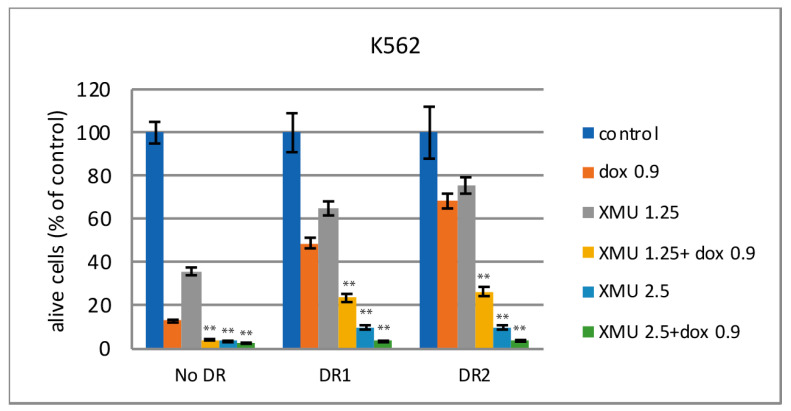
XMU-MP-1 efficiency in reverting the doxorubicin resistance in the K562 cells. Cells with no drug resistance (NoDR) and two cell sublines with drug resistance to doxorubicin (DR1 and DR2) were treated with XMU-MP-1 (1.25 or 2.5 µM) and doxorubicin (0.9 µM) during 8 days. DMSO alone was added to the control group. The plot shows % of live cells relative to the control. The plots show the mean ± SEM for three independent experiments. *t*-tests were performed, and asterisks indicate *p*-values relative to the only doxorubicin doxorubicin-treated cells (** *p* < 0.01).

As it can be seen from Figure 3, development of doxorubicin resistance increases cells’ resistance to XMU-MP-1. This indicates the development of resistance to different drugs in K562 cells. Noteworthy, their combined action suppresses resistance to both drugs. This effect may be accounted for by the fact that these two drugs have essentially different mechanisms of action, and the cell does not have enough time to develop drug resistance and overcome two very different suppression mechanisms at the same time.

## 3. Discussion

Cell signaling pathway inhibitors that control cell proliferation and death can be used in the complex therapy of malignant tumors. A properly chosen signaling pathway inhibitor can perform several functions in a pharmaceutical composition—it can enhance the efficiency of antitumor component activity against tumor cells, including by suppressing drug resistance development, and protect the body from the side effects of cytostatic. The efficiency of treatment depends on many factors such as, for example, the choice of drug dose, treatment regimen, etc. Suboptimal selection of parameters when using signaling pathway inhibitors may lead to an opposite effect: instead of inhibiting tumor growth, they may promote it. Therefore, it is important to look for the optimal combinations of chemotherapy and signaling pathway-affecting drugs and to expand the repertoire of drugs for antitumor therapy, especially for those tumors which cells are resistant to traditional cytostatics.

In the present work, we have shown that treatment with XMU-MP-1, the inhibitor of the MST1/2 kinase in the Hippo signaling pathway, prevents drug resistance development in the lymphoma and chronic myeloid leukemia cell lines and restores the sensitivity of resistant cells to doxorubicin.

A factor of XMU-MP-1 efficiency in combination with doxorubicin may be that it intervenes with the mechanisms involved in multidrug resistance development. The mechanisms of multidrug resistance observed in different hematological neoplasms may include overexpression of the ABC transporter protein genes, intensified drug metabolism due to altered molecular targets, defects in apoptosis, cell cycle disruption, glutathione-S-transferase overexpression, resistance to the microenvironment, and/or more efficient DNA repair mechanisms [16,17]. Resistance of cancer cells to doxorubicin may be linked to the inhibition of ferroptosis. SH3GL1-activated FTH1 inhibits ferroptosis and confers doxorubicin resistance in the diffuse large B-cell lymphoma [18]. STAT3 mediates multidrug resistance in the Burkitt’s lymphoma cells by promoting an antioxidant feedback mechanism [19].

Whole-transcriptome analysis of the Namalwa cells treated with XMU-MP-1, which we performed earlier [14], did not reveal any significant decrease in the ABC transporter mRNAs expression, including ABCC1(MRP1), ABCC2(MRP2), MVP, and ABCG2(BCRP). It is likely that the combination of XMU-MP-1 + doxorubicin may inhibit resistance development in hematological tumor cells through the mechanisms that are different from those involving the ABC transporter genes. Although, it cannot be excluded that the effects these compounds exert on the Hippo signaling pathway may extend to post-transcriptional modifications of ABC transporters, which reduce their activity [20].

We suggest that drug resistance suppression by XMU-MP-1 could be associated with downregulation of such metabolic genes involved in the metabolism of drugs and toxins as Glutation S-transferase (GST) and Digidrofolate reductase (DHFR). Owing to its role in detoxification, the GST gene has clinical significance in metabolic and pathological processes connected to treatment with xenobiotics. If GST is present at high levels, it neutralizes alkylating agents before they can damage cancer cell DNA. We have previously shown using RNA-Seq that mRNA levels for the GSTM1 and GSTZ1 glutathione-S-transferase genes as well as the Digidrofolate reductase (DHFR) gene are by several times decreased in the Namalwa cells in the presence of XMU-MP-1 (GEO database: GSE80287) [14].

To overcome drug resistance, chemotherapy should trigger the “suicide program” in the cell. We have previously demonstrated that XMU-MP-1 increases the expression of apoptosis activator genes in Namalwa cells, including APAF1, caspases 6 and 7, and the Fas death receptor on the cell surface; BBC3, BCL2A1, and MOAP1, which interacts with BAX and activates caspase-dependent apoptosis; XAF1, and other important regulators of apoptosis, which leads to the apoptotic death of Namalwa cells (GEO database: GSE80287) [14].

The suppression of drug resistance observed when the two drugs act together may be associated with the phenomenon of mitotic catastrophe. Mitotic catastrophe is one of the defense mechanisms that senses mitotic damage and prevents the survival and/or reproduction of cells with mitotic errors. Induction of mitotic catastrophe seems to be a promising strategy for increasing the sensitivity of tumor cells to chemotherapy. Usually, mitotic catastrophe is caused by low doses of chemotherapeutic agents, which are not enough to cause quick cell death [21]. The characteristic biochemical sign of mitotic catastrophe is mitotic arrest and the presence of multinucleated polyploid cells. This state is considered to correspond to the preliminary stage of cell suicide via apoptosis, necroptosis, or autophagy [21]. Mitotic catastrophe can result in the elimination of cells with defective mitosis and genomic instability, thereby preventing carcinogenesis. However, some cells may avoid mitotic catastrophe, which results in their premature exit from mitosis and transition to interphase.

In hematological tumor cells, XMU-MP-1 like doxorubicin causes mitotic catastrophe. We have previously shown that XMU-MP-1 triggers a cascade of transcription repression of the key cell cycle regulators in Namalwa cells (GEO database: GSE80287). XMU-MP-1 significantly downregulated the expression of cyclin B1 (CCNB1) and cyclin B2 (CCNB2), which are indispensable for cell cycle control at the G2/M (mitosis) transition, as well as other key cell cycle regulation genes, which led to the arrest of mitosis in the G2/M phase and activation of apoptosis [14]. At the same time, doxorubicin blocks the cell cycle at the G1/S stage.

We suggest that the combination of XMU-MP-1 and doxorubicin, which enhances the individual cytotoxic effects of doxorubicin and XMU-MP-1, increases the probability of the cell’s “non-return” and its death by inhibiting the key regulators of cell cycle. This may lead to the suppression of drug resistance development. However, this hypothesis needs further investigation.

Another mechanism, which may account for the enhanced effect of doxorubicin when combined with XMU-MP-1, may have relation to the fact that in hematological tumors, DNA damage leads to the activation of a proapoptotic pathway associated with the nuclear relocation of the ABL1 kinase. Previous studies have shown that low cellular levels of YAP1 can block the ABL1-induced apoptosis in hematological malignancies. At the same time, genetic inactivation of MST1 restores YAP1 levels causing cell death both in vitro and in vivo [22]. Hence, the simultaneous use of the two tested compounds can increase cell death rate in the Namalwa cells.

If XMU-MP-1 is able to prevent or delay the development of drug resistance, as our findings indicate, it should become an essential drug in the cytotoxic arsenal.

One more advantage of using the cytostatic + XMU-MP-1 combination is determined by XMU-MP-1 having a dual effect. On the one hand, XMU-MP-1 potentiates the effects of doxorubicin on hematological tumors and thereby makes it possible to reduce the effective dose of the cytostatic. On the other hand, XMU-MP-1 mitigates the toxic effects of chemotherapy and radiation therapy. Pretreatment with XMU-MP-1 significantly reduced the damage caused to the small intestine by total body irradiation with a dose of 9 Gy, increased the average survival time in mice exposed to the lethal radiation dose, and restored the impaired function of hematopoietic stem cells after total body irradiation [12]. XMU-MP-1 stimulates platelet recovery [13], alleviates the cytotoxic effects of paclitaxel on hair follicle cells [23], activates liver regeneration in humans [6], and improves cardiomyocyte survival and preserves cardiac function, which results in lower fibrosis levels [10].

It is possible that the combined use of doxorubicin and XMU-MP-1 may reduce the effective dose of doxorubicin used in the treatment of hematological cancers and thereby significantly reduce its life-threatening toxic effects such as free radical formation, which underlies the drug’s cardiotoxicity, thrombocytopenia, leukopenia, and necrotizing colitis. Our previous studies have also shown that XMU-MP-1 enhances the antitumor activity of other chemotherapeutic drugs such as etoposide and cisplatin towards Burkitt’s lymphoma cells [24]. Thus, XMU-MP-1 can potentially become an effective component in the combination therapy of hematological tumors.

Cytotoxic activity against hematological tumors and the ability to overcome drug resistance allow us to consider XMU-MP-1 as a promising candidate drug in combination with anthracyclines for the treatment of hematological tumors, especially drug-resistant ones.

This is the first report on the ability of XMU-MP-1 to prevent drug resistance development. Clinical implications of these findings are of huge importance. They suggest that anthracycline-sensitive tumors should apparently be treated with XMU-MP-1 from the very outset to get maximum benefit from the combination of these drugs.

## 4. Materials and Methods

### 4.1. Cell Lines

Human cell lines: B-cell lymphoblastoid Burkitt’s lymphoma Namalwa, T-cell lymphoblastoma Jurkat, and human chronic myeloid leukemia K562 were obtained from the Russian Cell Culture Collection, Institute of Cytology, St. Petersburg, Russia. Cells were maintained in DMEM (GIBCO, Thermo Fisher Scientific, Waltham, MA, USA) with 10% FCS (FBS; HyClone, Logan, UT, USA), 100 U/mL penicillin, and 100 μg/mL streptomycin, in 5% CO_2_.

### 4.2. Cell Cycle Assay with Flow Cytometry (FCM)

Cells were inoculated into 60 mm^2^ dishes and treated with either 1.25 µM XMU-MP-1, or 0.6 µM doxorubicin, or 1.25 µM XMU-MP-1 + 0.6 µM doxorubicin. Only DMSO was added to the control group. Measurements were taken in three sessions. Furthermore, 24 h and 48 h after the treatment, treated cells were fixed in 70% ethanol and stored at −20 °C. For the *FCM* assay, frozen cells were washed with PBS, stained with 50 µg/mL propidium iodide (PI), and treated with 100 µg/mL RNase A (Sigma, cat. no. R-4875) at 37 °C for 60 min. Cells were analyzed in the BD LSRFortessa Cell Analyzer using the provided BD Bioscience software (Becton Dickinson, San Jose, CA, USA). Cells were distributed between the G1, S, G2/M, and sub-G1 subpopulations based on their fluorescence.

### 4.3. In Vitro Cell Viability Assay

To test the cytotoxicity of mono- and combined treatments, cells were inoculated into 96-well plates at the density of 30,000 (Namalwa) or 10,000 (K562) cells per well in 100 µL of the DMEM medium (PanEco, Moscow, Russia) supplemented with 10% fetal calf serum (BioSera, Cholet, France). Drug combinations were added to the wells. Cells were cultured in the cultural plates (TRP, Zurich, Switzerland) in DMEM (PanEco, Russia) at +37 °C in 5% CO_2_. XMU-MP-1 cytotoxicity was assessed using the CellTiter 96 AQueous One Solution kit (Promega, Madison, WI, USA). Antiproliferative activity of XMU-MP-1, doxorubicin, and XMU-MP-1 + doxorubicin was studied by treating cells with XMU-MP-1 at the concentrations of 0.6; 1.25, and 2.5 μM, doxorubicin, at 0.0375; 0.075; 0.15; 0.3; 0.6; and 0.9 µM, and their combinations (at least three replicates for each concentration). Cells grown only with DMSO and no tested compounds added served as the control. After 48 and 72 h of incubation, antiproliferative and cytotoxic effects of XMU-MP-1 were analyzed according to the manufacturer’s protocol. The optical density of solutions was measured at 490 nm using the Chameleon V plate reader (Hydex Oy, Turku, Finland). The amount of the formazan product calculated based on the absorbance rate at 490 nm is in the direct proportion to the number of live cells in the culture.

### 4.4. In Vitro Model System to Study Drug Resistance Development

Optimal concentrations for long-term cultivation were chosen for each drug and their combinations. Cells were inoculated into the 25 cm flasks in the amount of 4 million cells per flask.

The following culturing options were used for the Namalwa cells:

1. Culturing for 48 h in the complete DMEM medium in the presence of XMU-MP-1 (Sigma) (0.625, 1.25, or 2.5 µM), then pelleting cells and resuspending them in the complete DMEM medium with added doxorubicin (0.15 µM or 0.3 µM), and then long-term culturing.
2. Culturing for 48 h in the complete DMEM medium containing XMU-MP-1 (0.625, 1.25, or 2.5 µM) followed by pelleting cells, resuspending them in the fresh medium, and adding XMU-MP-1 (0.625, 1.25, or 2.5 µM).
3. Culturing for 48 h in the complete DMEM medium containing DMSO alone followed by pelleting cells, resuspending them in the fresh medium, and adding doxorubicin (0.15 µM or 0.3 µM).
4. The control cells were cultured in the complete DMEM medium containing only DMSO and no tested compounds. The control cells were passaged 1/10 every 5 days.

The following culturing options were used for the K562 cells:

1. Culturing for 96 h in the complete DMEM medium in the presence of XMU-MP-1 (Sigma) (1.25 or 2.5 µM), then pelleting cells and resuspending them in the fresh medium containing XMU-MP-1 (1.25 or 2.5 µM) + doxorubicin (0.6 µM or 0.9 µM), and then long-term culturing.
2. Culturing for 96 h in the complete DMEM medium containing XMU-MP-1 (1.25 or 2.5 µM) followed by pelleting cells and resuspending them in the fresh medium containing XMU-MP-1 (1.25 or 2.5 µM).
3. Culturing for 96 h in the complete DMEM medium with DMSO alone, followed by pelleting cells and resuspending them in the fresh medium containing doxorubicin (0.6 µM or 0.9 µM).
4. Culturing in the complete DMEM medium containing only DMSO and no tested compounds. The control cells were passaged 1/10 every 5 days.

Medium was changed every 5 days. Cell chemosensitivity was analyzed by direct cell counting after trypan blue staining using a microscope. The time point when the survived cells reach the initial density of 2 × 10^6/8^ mL (so-called “repopulation”) was recorded. Toxicity of different concentrations and combinations of the tested compounds was determined based on cells’ viability relative to the control.

### 4.5. Assessment of the Efficiency of Overcoming Doxorubicin Drug Resistance

To test whether XMU-MP-1 can act as a drug resistance modulator in the short term, two K562 sublines (DR1 and DR2) resistant to doxorubicin at the concentration of 0.6 µM were obtained.

The original K562 cells with no drug resistance (NoDR) and the derived sublines (DR1 and DR2) were inoculated into the 25 cm flasks in the amount of 4 million cells per flask and treated with 0.9 µM doxorubicin, as indicated above, in combination with XMU-MP-1 (1.25 or 2.5 µM) or without XMU-MP-1 for 8 days. Cells from each subline that were not treated with doxorubicin and XMU-MP-1 were used as the controls.

Cell chemosensitivity was then analyzed by direct cell counting after trypan blue staining using microscope. Drug resistance of cells in the presence of different concentrations and combinations of the tested compounds was estimated based on cells’ viability relative to the control.

### 4.6. Statistics

All experiments were repeated at least three times. Statistical analysis of the cell proliferation assay and flow cytometry assay results was conducted using the GraphPad software v10 (GraphPad Software, San Diego, CA, USA). Student’s *t*-tests were used to generate *p*-values. One-way analysis of variance (ANOVA) followed by Dunnett’s test was used for multiple comparisons. Values of *p* < 0.05 were considered significant, and values of *p* < 0.01 were considered extremely significant.

## 5. Conclusions

This work provides the first evidence that XMU-MP-1, the Hippo signaling pathway MST1/2 kinase inhibitor, prevents or significantly inhibits the development of drug resistance in lymphoma and a chronic myeloid leukemia cell lines. These effects suggest that XMU-MP-1 has potential to enhance the efficiency of doxorubicin in the treatment of hematological tumors. Its cytotoxic activity and the ability to overcome drug resistance allow considering XMU-MP-1 as a promising candidate drug for antileukemic therapy.

## Data Availability

The original contributions presented in the study are included in the article and Supplementary Material, further inquiries can be directed to the corresponding author. (GEO database: GSE80287)

## Supplementary Materials

The following supporting information can be downloaded at: https://www.mdpi.com/article/10.3390/ph19071075/s1, Figure S1: Morphological changes in K562 cells after cultivation in the presence of 1.25 μM XMU-MP-1 or 1.25 μM XMU-MP-1 + 0.6 μM doxorubicin for 5 days; Figure S2: XMU-MP-1 reduces the amount of phosphorylated MST1 in cells. Table S1: *p*-values were calculated using Welch’s *t*-test for the Namalwa andK562 cell groups treated with (XMU-MP-1 + DOX) or only XMU-MP-1 or DOX; Table S2: Effect of XMU-MP-1 on doxorubicin IC50.
