# Supplementary material for: XMU-MP-1, the Hippo Signaling Pathway MST-1 Kinase Inhibitor, Prevents the Development of Drug Resistance to Doxorubicin in Hematological Tumor Cells
Source: Pharmaceuticals (Basel). 2026 Jul 12;19(7):1075. doi: 10.3390/ph19071075 (PMC13414586; doi:10.3390/ph19071075)

Supplementary materials (Stepchenko A.G., Pankratova E.V)

XMU-MP-1, the Hippo Signaling Pathway MST-1 Kinase Inhibitor, Prevents the Development of Drug Resistance to Doxorubicin in Hematological Tumor Cells

Figure S1.

K562 <u>Hoechst</u> <u>phase contrast</u> <u>merge</u>

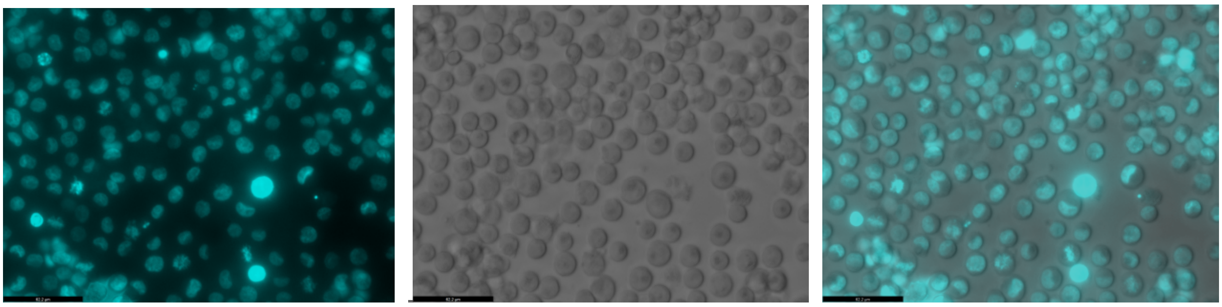

K562+XMU-MP-1 (1.25 μM)

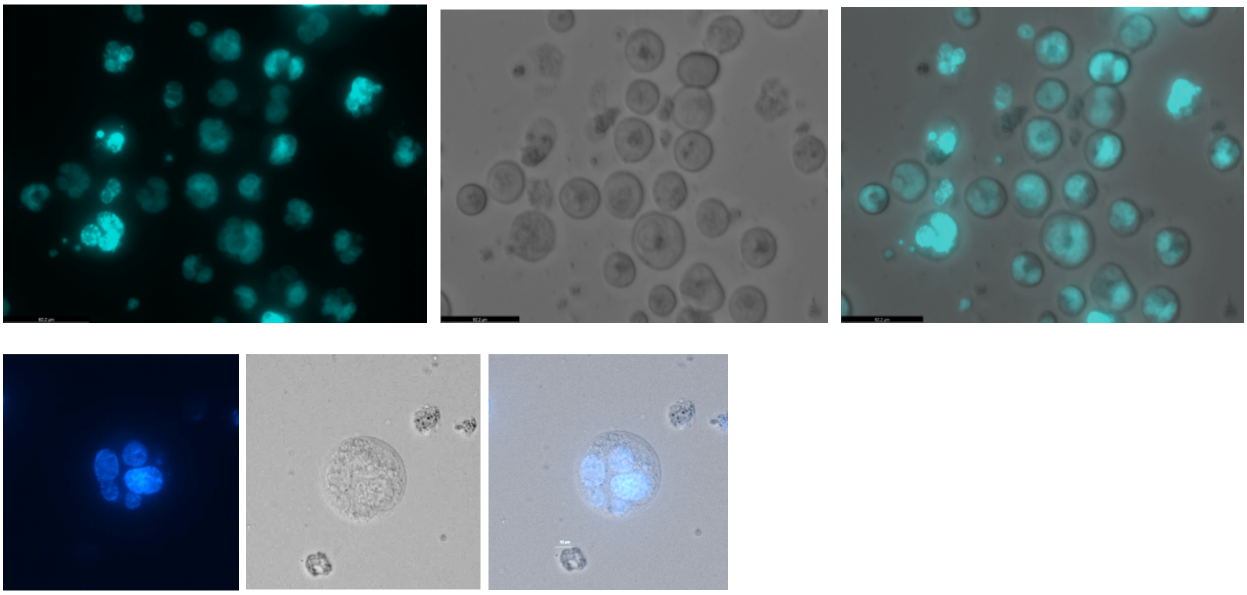

K562+ XMU-MP-1 (1.25 μM)+DOX (0.6 μM)

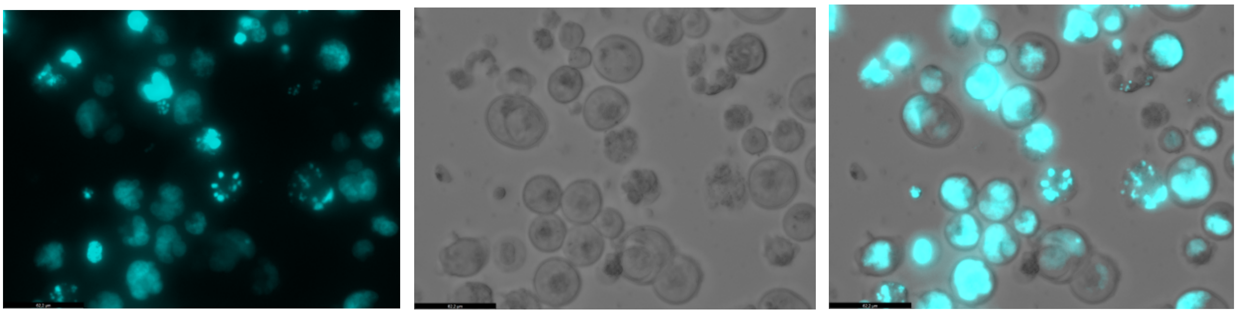

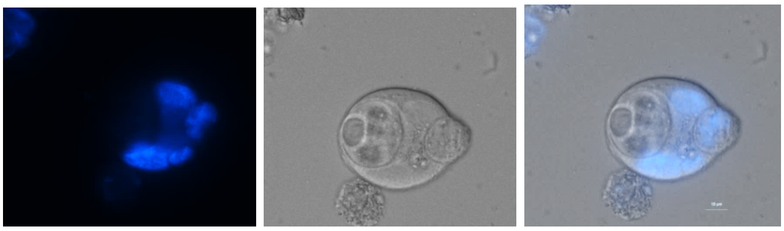

Figure S1. Morphological changes in K562 cells after cultivation in the presence of 1.25 μM XMU-MP-1 or 1.25 μM XMU-MP-1 + 0.6 μM doxorubicin for 5 days. Nuclear staining with Hoechst 33342 (Thermo Fisher Scientific), according to the manufacturer's instructions.

Figure S2.

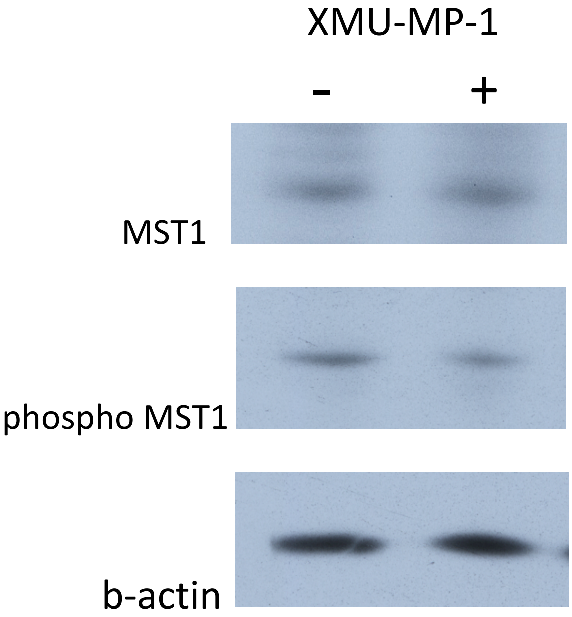

Figure S2. XMU-MP-1 reduces the amount of phosphorylated MST1 in cells. Western blot hybridization. Cell extracts from K562 cells cultured in the presence of 1.25 μM XMU-MP-1 for 24 hours and from control cells were prepared using a buffer containing 50 mM Tris-HCl pH 7.5, 150 mM NaCl, 1% Triton X-100, 1 mM EDTA, 1 mM PMSF, and phosphatase inhibitor cocktail (Sigma-Aldrich, Germany). Protein concentration was determined using the BCA Protein Assay Kit (ThermoFisher Scientific). After centrifugation (10,000 rcf, 10 min, +4°C), the loading buffer was added to the supernatants, heated at 95 ^{0}C, 5 min and used for electrophoresis in 8% acrylamide gel and for Western blotting according to the antibody manufacturer's protocol. The following primary antibodies were used: rabbit polyclonal to MST-1 diluted 1:1000 (3682) (Cell Signaling), rabbit monoclonal to phospho-MST1 (Thr 183/180) clone 1C2 diluted 1:1000 (ZRB2396) (Sigma-Aldrich), rabbit monoclonal to beta-actin diluted 1:1000 (13E5) (Cell Signaling). Finally, the membranes were stained with standard ECL reagents and autographed.

Table S1. P-values were calculated using Welch's t-test for the Namalwa and K562 cell groups treated with (XMU-MP-1 + DOX) or only XMU-MP-1 or DOX.

Namalwa

days	7	11	14	16	18	20	22	27	30	33	38	40
P-value (XMU) – (XMU + DOX)	0.082	0.101	0.09	0.103	0.023	0.019	0.026	0.034	0.041	0.019	0.028	0.022
P-value (DOX) – (XMU + DOX)	0.007	0.001	0.09	0.061	0.027	0.018	0.021	0.009	0.007	0.001	0.016	0.017

K562

days	4	7	13	17	26	31	37	41	48	53
P-value (XMU) – (XMU + DOX)	0.104	0.076	0.005	0.031	0.023	0.024	0.012	0.028	0.027	0.013

Table S2. Effect of XMU-MP-1 on doxorubicin IC50.

Cells	DOX IC50 (µM)		Sensitization ratio (DOX IC50/ (DOX+XMU) IC50)
	- XMU-MP-1	+ XMU-MP-1 (2.5 µM)	
Parental (No DR)	0.52 ± 0.13	0.22 ± 0.03	2.36
Resistance (DR1)	6.84 ± 0.47	0.52 ± 0.1	13.1
Resistance (DR2)	8.69 ± 0.38	0.61 ± 0.18	14.2

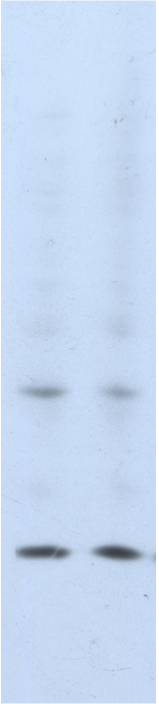

Supplement: Supplementary file 1 [file pharmaceuticals-19-01075-s001.zip › pharmaceuticals-4313448-supplementary.pdf]
